# Not All Grains Are Created Equal: Gluten-Free Products Not Included in Mandatory Folate Fortification

**DOI:** 10.1093/cdn/nzz020

**Published:** 2019-03-27

**Authors:** Stephanie Mitchell, Allison Gomes, Rena Zelig, Anna Parker

**Affiliations:** School of Health Professions, Department of Clinical and Preventive Nutrition Sciences at Rutgers University, Newark, NJ

**Keywords:** folate, folic acid fortification, gluten free, gluten allergy, gluten intolerance, neural tube defects

## Abstract

Adequate folate intake during the female reproductive years plays a vital role in fetal neurodevelopment. To address this public health concern, the FDA required enriched cereal grains to be fortified with folic acid. A positive outcome of fortification with folic acid was a reduction in the number of pregnancies affected by neural tube defects (NTDs). However, there are individuals unable to consume these enriched grains, such as those with a gluten allergy or intolerance. The need for folic acid fortification across all grains, those with and without gluten, should be considered in an effort to provide equivalent folic acid to all and further promote public health efforts aimed at decreasing the incidence of NTDs.

Folate is a micronutrient that is required for DNA synthesis and as a substrate for various enzymatic reactions involved in amino acid synthesis and vitamin metabolism (1). For females of reproductive age, the role of folate in cell growth and DNA production is crucial because it is involved in reproducing important neurological cells vital to the development of the brain and spine of the fetus (2). Without adequate intake of folate, ≥400 µg folate/d during reproductive ages, women are at risk of having a pregnancy affected by abnormalities such as megaloblastic anemia, peripheral neuropathy, or congenital abnormalities in the fetus, including neural tube defects (NTDs) (1–3). The 2 most common NTDs are spina bifida and anencephaly, both of which occur in early pregnancy and result when the spine and brain, respectively, do not close appropriately (4). After the FDA required fortification of enriched grains with folic acid, specifically cereal grains, there was a decline of >25% in the number of pregnancies affected by NTDs from prefortification (1995–1996) to postfortification (1999–2000) (5). Grains that are required to be fortified with folic acid in the United States are listed in **Table 1**; however, this requirement does not cover all grains, and the majority of gluten-free grains do not fall under this requirement, placing women of reproductive age who follow a gluten-free diet at risk of inadequate intake of folic acid (**Table 2**).

**TABLE 1:** Grains required to be fortified with folic acid in the United States^1^

Breakfast cereals made from wheat, *corn*, *oats*,^2^ and *rice*
Enriched cereal grain products
Wheat flour–based products
Triticale
Farina
Bulger
Semolina
Durum
Wheat germ
*Rice—whole kernel*
*Cornmeal*
*Corn grits*

^1^Source: references 6 and 7. Italicized grains are gluten free.

^2^Oats are constitutionally gluten free but can be cross-contaminated with wheat during processing.

**TABLE 2:** Examples of gluten-free grains not required to be fortified with folic acid in the United States^1^

Amaranth
Buckwheat
Quinoa
Rice flour
Oat flour^2^
Brown rice flour
White rice flour (made from non-enriched white rice)
Tapioca flour
Nut-based flours (e.g., almond flour)
Potato flour
Teff
Sorghum
Wild rice
Corn masa flour^3^

^1^Source: references 6–8, 13, 16.

^2^Oats are constitutionally gluten free but can be cross-contaminated with wheat during processing.

^3^Voluntary fortification as of April, 2016.

Multiple studies supported the benefit of folic acid supplementation in the prevention of NTDs, even before its establishment as a public health concern in the early 1990s (8–11). One of these landmark studies in 1991 was a multicenter randomized prevention trial completed by the British Medical Research Council Vitamin Study Group (8). Researchers assessed the role of folic acid supplementation among women with a history of a pregnancy affected by an NTD (*n* = 1817) (8). The women were randomly assigned into 1 of 4 supplementation groups (4 mg folic acid, multivitamin with 4 mg folic acid, a multivitamin without folic acid, and no supplementation). Of the women who became pregnant during the study (*n* = 1195), 21 of the 27 pregnancies affected by an NTD were in mothers randomly assigned to the groups without folic acid supplementation (8). The researchers concluded that all women of reproductive age should consume ≥400 µg folic acid/d preconceptually and during pregnancy to reduce the risk of an NTD-affected pregnancy.

After the association between folic acid intake and reduction of NTDs was well known in the medical field, a study conducted by Forman et al. (12) in 1996 assessed the periconceptual intake

of folic acid among Canadian women that gave birth to children with spina bifida. The results revealed that despite living in an advanced country where the association between folate and NTDs was known, only 13% of the women studied were aware that this relation existed, and those who did know did not alter their dietary intake of foods rich in folate to reduce their risk of having a pregnancy affected by NTDs (12). This public health issue resulted in the 1996 finalized ruling by the FDA mandating that enriched cereal grains be fortified with folic acid (140 µg/100 g) within the next 2 y (6). Cereal grains were chosen based on the success of previous fortification and consumption patterns of the intended target population (6).

The result of mandated folic acid fortification was the reduction in annual deaths related to NTDs from 1200 prefortification (1995–1996) to 840 postfortification (1999–2000) and a 27% decline in the number of pregnancies affected by NTDs pre- to postfortification (5). Although the fortification of enriched grains with folic acid has been effective at reducing the incidence of infants born with an NTD, not all grains are required to be fortified with folic acid (Tables 1 and 2). The FDA expanded fortification to allow for corn masa flour to be fortified to an equivalent level of other enriched grains, yet this expansion was on a voluntary basis beginning in April, 2016 (13).

The incidence of individuals following gluten-free diets in the United States has been increasing significantly since 2009, despite the fact that celiac disease (CD) prevalence has remained stable (14). CD is an autoimmune disease that affects the gastrointestinal tract. When an individual with CD consumes gluten-containing foods, it causes inflammation in the gut and leads to eventual atrophy of the villi within the intestines and malabsorption of nutrients. Therefore, for disease remission, those with CD must avoid gluten-containing grains (15) and wheat-, barley-, and rye-derived flours and use alternative flours such as oat- or nut-based flours. With the exception of rice flour made from enriched white rice or corn masa that is voluntarily fortified, many of the gluten-free alternative flours are not required to be fortified with folic acid (13, 16). The number of individuals following a gluten-free diet is ∼1% of the US population or 3.1 million people, many of whom are female (14). The number of women who follow a gluten-free diet despite having no medical reason is likely due to the influence of social and other media on diet for weight loss and/or various definitions of a “healthy diet.” These women are not getting the benefit of enriched grain products fortified with folic acid, which may lead to an increase in the incidence of NTDs which has not otherwise occurred since the initial fortification of enriched grain products in 1998.

The Canadian Food Inspection Agency has put forth a regulation that encourages gluten-free products to be fortified to meet the standards of their gluten-containing food product counterparts. This is in contrast to many European countries where fortification with folic acid, in gluten-containing and gluten-free grains, is not mandatory (17, 18). Other than corn masa flour, there are no regulations, voluntary or mandatory, for the fortification of gluten-free products in the United States (13). In fact, many people incorrectly assume that gluten-free grains are fortified to the level of gluten-containing grains. This misunderstanding regarding folic acid fortification of gluten-free products can result in the unintentional under-consumption of folic acid by women of childbearing age following a gluten-free diet, placing them at increased risk of a pregnancy affected by an NTD. Given the benefits of folic acid fortification and the risks that accompany inadequate intake of folic acid during pregnancy, there is a need to fortify gluten-free products in this country.

As registered dietitians, and as people who work with or personally deal with serious gluten allergy and intolerance, we implore that the US government and FDA consider the need for regulations to fortify all gluten-free flour products with folic acid in the United States. All grain products, regardless of whether or not they contain gluten, should be fortified in an effort to provide equivalent folate to all who consume these products. This will help to continue to promote public health efforts to decrease the incidence of NTDs.

## Notes

The authors reported no funding received for this study.

Author disclosures: SM, AG, RZ, and AP, no conflicts of interest.
